# Regulation of Vascular Endothelial Integrity by Mesenchymal Stem Cell Extracellular Vesicles after Hemorrhagic Shock and Trauma

**DOI:** 10.21203/rs.3.rs-4284907/v1

**Published:** 2024-05-02

**Authors:** Mark Barry, Alpa Trivedi, Byron Miyazawa, Lindsay Vivona, David Shimmin, Praneeti Pathipati, Callie Keane, Joseph Cuschieri, Shibani Pati

**Affiliations:** University of California San Francisco; University of California San Francisco; University of California San Francisco; University of California San Francisco; University of California San Francisco; University of California San Francisco; University of California San Francisco; University of California San Francisco; University of California San Francisco

**Keywords:** mesenchymal stem cells, mesenchymal stem cell extracellular vesicles, hemorrhagic shock, trauma

## Abstract

**BACKGROUND:**

Patients with hemorrhagic shock and trauma (HS/T) are vulnerable to the endotheliopathy of trauma (EOT), characterized by vascular barrier dysfunction, inflammation, and coagulopathy. Cellular therapies such as mesenchymal stem cells (MSCs) and MSC extracellular vesicles (EVs) have been proposed as potential therapies targeting the EOT. In this study we investigated the effects of MSCs and MSC EVs on endothelial and epithelial barrier integrity *in vitro* and *in vivo* in a mouse model of HS/T. This study addresses systemic effects of HS/T on multiorgan EOT in HS/T model.

**METHODS:**

*In vitro*, pulmonary endothelial cell (PEC) and Caco-2 intestinal epithelial cell monolayers were treated with control media, MSC conditioned media (CM), or MSC EVs in varying doses and subjected to a thrombin or hydrogen peroxide (H_2_O_2_) challenge, respectively. Monolayer permeability was evaluated with a cell impedance assay, and intercellular junction integrity was evaluated with immunofluorescent staining. *In vivo*, a mouse model of HS/T was used to evaluate the effects of lactated Ringer’s (LR), MSCs, and MSC EVs on endothelial and epithelial intercellular junctions in the lung and small intestine as well as on plasma inflammatory biomarkers.

**RESULTS:**

MSC EVs and MSC CM attenuated permeability and preserved intercellular junctions of the PEC monolayer *in vitro*, whereas only MSC CM was protective of the Caco-2 epithelial monolayer. *In vivo*, both MSC EVs and MSCs mitigated the loss of endothelial adherens junctions in the lung and small intestine, though only MSCs had a protective effect on epithelial tight junctions in the lung. Several plasma biomarkers including MMP8 and VEGF were elevated in LR- and EV-treated but not MSC-treated mice.

**CONCLUSIONS:**

In conclusion, MSC EVs could be a potential cell-free therapy targeting endotheliopathy after HS/T via preservation of the vascular endothelial barrier in multiple organs early after injury. Further research is needed to better understand the immunomodulatory effects of these products following HS/T and to move toward translating these therapies into clinical studies.

## BACKGROUND

Patients with severe injuries and hemorrhagic shock are susceptible to the endotheliopathy of trauma (EOT), which includes vascular barrier compromise, systemic inflammation, and dysfunctional coagulation.^1–3^ The EOT is closely linked with multiple organ failure and contributes to increased mortality after injury.^4,5^ While plasma-based resuscitation and modern trauma systems have improved outcomes in severely injured patients,^6,7^ trauma remains a leading cause of death in the United States,^8^ and novel therapies are needed.

Bone marrow derived mesenchymal stem cells (MSCs) are known to have vasculoprotective, anti-inflammatory, and regenerative properties and are the focus of numerous ongoing clinical trials.^9,10^In light of these properties, MSCs have been proposed as a potential therapy targeting the EOT and have been shown to reduce injury in multiple organs^11–14^ and reverse bone marrow suppression^15^ in rodent models of hemorrhagic shock and trauma (HS/T). Though initially MSCs were thought to act through their ability to home to injured tissue, the majority of intravenously administered MSCs become trapped in capillary networks such as in the lungs; one of their primary mechanisms of action is now understood to be through the release of bioactive soluble factors and extracellular vesicles (EVs).^10,16,17^ EVs are membrane-bound particles containing RNA, DNA, protein, and lipids from their parent cells and are highly effective mediators of intercellular communication.^18^ MSC EVs have been proposed as a “cell-free” therapy as they have been shown to recapitulate the therapeutic properties of MSCs.^17,19^

We recently demonstrated in a mouse model of HS/T that both MSCs and MSC EVs mitigate histologic injury and vascular permeability to a 10 kDa dextran dye in the small intestine and lungs.^12^ The purpose of the current study was to further investigate the molecular mechanism of MSCs and MSC EVs in HS/T, specifically their effects on endothelial and epithelial barrier integrity and on systemic inflammatory biomarkers. We hypothesized that MSCs and MSC EVs would (1) preserve pulmonary endothelial and intestinal epithelial barrier integrity *in vitro* and (2) preserve intercellular junctions in the small intestine and lungs and reduce plasma inflammatory biomarker levels *in vivo*.

## METHODS

### Mesenchymal Stem Cell Culture and EV Isolation

Human bone marrow-derived MSCs (passage 1) were obtained from Rooster Bio Inc. (Frederick, MD) and expanded initially on a Terumo Quantum Device (Terumo, Lakewood, CO) to generate passage 2 cells that were used in all studies. MSCs were grown in Mesenchymal Stem Cell Growth Medium 2 (PromoCell, Heidelberg, Germany) and maintained at 37°C and 5% CO2 in a humidified incubator. To isolate MSC EVs, the MSCs were grown to 80% confluence, then serum-starved for 48 hours. The MSC conditioned media (CM) was collected and centrifuged at 1500g × 10 minutes to remove cellular debris, then filtered using a 0.22μm filter. EVs were isolated from the MSC CM using a Tangential Flow Filtration System with the Pellicon^®^ XL50 Cassette with Biomax^®^ 500 kDa Membrane (MilliporeSigma, Burlington, MA). A subset of the isolated EVs were further concentrated using an Amicon Ultra-2 Centrifugal Filter 3K Device (MilliporeSigma) according to manufacturer instructions for use in the *in vitro* experiments. Aliquots of isolated EVs were stored at −80°C. The MSC EVs used in this study were previously characterized by flow cytometry, nanoparticle tracking analysis, and spectrophotometry as previously described,^12^ and scanning electron microscopy images were also captured for this study (**Supplemental Figure 1**).

### Pulmonary Endothelial Cell (PEC) and Caco-2 Intestinal Epithelial Cell Culture

Human pulmonary microvascular endothelial cells (PECs) were obtained from PromoCell and grown in Endothelial Cell Growth Medium MV2 (PromoCell). Passages 3–7 were used in all experiments. Caco-2 human intestinal epithelial cells were obtained from American Type Culture Collection (ATCC, Manassas, VA) and were grown in Eagle’s Minimum Essential Medium (ATCC) supplemented with 20% Fetal Bovine Serum and 1% penicillin/streptomycin. Passages 2–5 were used in all experiments. Both cell lines were maintained at 37°C and 5% CO2 in a humidified incubator

### In Vitro Pulmonary Endothelial Cell (PEC) Monolayer Barrier Integrity and Intercellular Junction Immunostaining

Pulmonary endothelial cell (PEC) monolayer barrier integrity was measured *in vitro* using an electric cell-substrate impedance sensing system (ECIS 1600, Applied BioPhysics, Troy, NY). To evaluate the PEC monolayer, PECs (50,000 cells per well) were seeded onto a 96-well plate containing electrodes that had been pre-treated with L-cysteine and were grown to confluence. The PEC monolayers were serum-starved for one hour and once resistances had stabilized were then pre-treated with MSC control media (10% or 20% v/v), MSC CM (10% or 20% v/v), or MSC EVs (10 or 30 μg/ml). After 30 minutes the PEC monolayers were challenged with thrombin at 0.2u/ml to induce paracellular permeability. Resistances were measured in 5-minute intervals at 4000 Hz. Data were normalized to the mean resistance of the monolayers before the treatments. Resistance tracings and area under the curve (AUC) plots were generated for each treatment group to compare monolayer integrity.

To evaluate intercellular junction integrity of the PEC monolayer, PECs were separately grown on cover slips (coated with collagen type 1) in 24-well plates (50,000 cells per well). The wells were pre-treated with MSC control media (10% or 20% v/v), MSC CM (10% or 20% v/v) or MSC EVs (30 μg/ml) for 30 minutes then challenged with thrombin 0.2u/ml. 5 min after the addition of thrombin, the cells were washed three times with PBS then fixed with 4% PFA. Immunostaining was then performed using antibodies against VE-cadherin (Cell Signaling, Danvers, MA) and Zonula Occludens-1 (ZO-1, Abcam, Burlingame, CA). F-actin was detected with Texas Red Phalloidin (Cell Signaling). Representative images were captured using a Nikon Eclipse 80i microscope (Nikon, Melville, NY) with an RT-scmos camera (SPOT Imaging, Sterling Heights, MI).

### In Vitro Caco-2 Intestinal Epithelial Cell Monolayer Barrier Integrity and Intercellular Junction Immunostaining

To evaluate the Caco-2 intestinal epithelial monolayer, Caco-2 cells (25,000 cells per well) were seeded onto a 96-well plate for ECIS as above and grown to confluence. The Caco-2 monolayers were serum-starved for 2 hours and once resistances had stabilized were then pre-treated with MSC control media (10% or 25% v/v), MSC CM (10% or 25% v/v), or MSC EVs (10, 30, or 50 μg/ml). After 30 minutes the Caco-2 cell monolayers were challenged with hydrogen peroxide (H_2_O_2_) at 2.5mM to cause oxidative stress; this dose was chosen based on preliminary studies in the lab showing decreased resistance across the monolayer in this model. Resistances were measured in 4-minute intervals at 1000 Hz. Data normalization, resistance tracings, and AUC plot generation were conducted as above.

To evaluate intercellular junction integrity of the Caco-2 monolayer, Caco-2 cells were separately seeded on to 24-well plates (80,000 cells per well). The cells were pre-treated with MSC control media (10% or 25% v/v), MSC CM (10% or 25% v/v) or MSC EVs (30 μg/ml) for 30 minutes then challenged with H_2_O_2_ 2.5mM. 2.5 hours after exposure to H_2_O_2_, the cells were washed three times with PBS then fixed with 4% PFA. Immunostaining was then performed using antibody against ZO-1 (Abcam), and F-actin was detected using Texas Red Phalloidin (Cell Signaling). Representative images were captured using a Revolve microscope (Echo Inc., San Diego, CA).

### Animal Studies

Animal studies were performed with approval of the Institutional Animal Care and Use Committee (IACUC) at UCSF. The experiments were conducted in compliance with the ARRIVE guidelines for animal models and the National Institutes of Health (NIH) guidelines on the use of laboratory animals. All animals were house in a room with access to food and water ad libitum, controlled temperature, and 12:12-hour light-dark cycles.

### Mouse Model of Hemorrhagic Shock and Trauma

Male C57BL6 mice, 8–12 weeks old, were obtained from The Jackson Laboratory (Sacramento, CA) (N=20 total). Mice underwent an established model of HS/T.^12,20,21^ Briefly, the mice were anesthetized with isoflurane and maintained at a body temperature between 35°C and 37°C using a heating plank. The bilateral femoral arteries were cannulated with heparinized catheters, one for continuous blood pressure monitoring (PowerLab 7, AD Instruments, Dunedin, New Zealand), and the other for blood withdrawal and resuscitation. A 2cm midline laparotomy was also performed to induce additional trauma. Mice were subsequently bled to a mean arterial pressure (MAP) of 35mmHg for 90 minutes and then resuscitated with a 200μL fluid bolus containing 1) lactated Ringer’s (LR), 2) 1 × 10^6^ MSCs in LR, or 3) MSC EVs (30μg) in PBS. These doses were chosen based on previous work demonstrating efficacy of MSC and MSC EVs in this model.^12,21^ Sham mice underwent cannulation without laparotomy or hemorrhage. Mice were monitored hemodynamically for an additional 30 minutes after resuscitation. Two hours post-resuscitation, the mice were re-anesthetized with isoflurane. Blood was collected via cardiac puncture and the mice were perfused with 10mL of ice-cold PBS. Sodium citrate 3.2% was added to the blood in a 1:9 ratio prior to centrifugation at 3000g for 10 minutes to isolate the plasma fraction, which was stored at −80°C. The lungs and a segment of small intestine were harvested and flash-frozen in isopentane and stored at −80°C.

### Intercellular Junction Immunostaining of the Small Intestine and Lungs

The lungs and small intestine from N=5 mice per group were sectioned at 10μm thickness. The sections were fixed in ice-cold 95% EtOH for 20 minutes then 100% acetone for 1 minutes. Immunostaining was then performed using antibodies against the adherens junction protein VE-cadherin (R&D Systems, Minneapolis, MN) and the tight junction proteins ZO-1 (Abcam, Burlingame, CA) and claudin-4 (Thermo Fisher, Waltham, MA). Sections were imaged in a blinded fashion with a Nikon Eclipse 80i microscope (Nikon) with an RT-scmos camera (SPOT Imaging), and representative images were selected from each animal for qualitative comparison.

### Plasma Inflammatory Biomarker Analysis

Mouse plasma samples (N=5 per group) were analyzed using a custom multiplex Luminex^®^ Discovery Assay Kit (R&D Systems, Minneapolis, MN) according to the manufacturer’s protocol. The following analytes were included: Angiopoietin-2 (Ang-2), C-X-C Motif Chemokine Ligand 10 (CXCL10), CXCL12, Intercellular Adhesion Molecule-1 (ICAM-1), Interferon gamma (IFN-γ), Interleukin-1beta (IL-1β), IL-2, IL-4, IL-6, IL-6 receptor alpha (IL-6Ra), IL-10, Matrix Metalloproteinase-8 (MMP8), MMP9, Syndecan-1, Tumor Necrosis Factor alpha (TNFα), TNF Receptor Superfamily Member 1a (TNFRSF1a), TNFRSF1b, TNF Superfamily Member 13b (TNFSF13b), and Vascular Endothelial Growth Factor (VEGF). Plasma samples were thawed and centrifuged at 16,000g for 4 min prior to use. Samples were then diluted 1:2 and 1:10 in appropriate diluent and pipetted onto a 96-well plate, mixed with magnetic beads coated with antibodies, and incubated for 2 hrs at room temperature on a horizontal orbital microplate shaker at 800rpm. Three washes with wash buffer were performed, then the beads were incubated with the biotinylated antibody cocktail for 1 hr at room temperature on the orbital shaker at 800rpm. After another three washes, the beads were incubated with streptavidin-PE for 30 minutes on the shaker. The beads were washed a final three times then resuspended in wash buffer prior to being read on a MAGPIX System (Luminex Corp., Austin, TX). xPONENT 4.2 software (Luminex Corp.) was used for data acquisition.

### Statistical Analysis

Area under the curve values for the *in vitro* experiments were compared using one-way ANOVA with Tukey’s post hoc tests. Mean arterial pressures (MAPs) were compared using repeated measures two-way ANOVA with Tukey’s multiple comparisons test. For the plasma biomarker analysis, first outliers were removed using the ROUT method in Prism 9.0 (GraphPad Inc., San Diego, CA) and data were assessed for normality using the Shapiro-Wilk test. One-way ANOVA with post-hoc Tukey’s test was used for normally distributed data, and Kruskal-Wallis testing with Dunn’s multiple comparisons test was used for data that did not pass the normality testing. P<.05 was considered significant. All analyses were performed using Prism 9.0. Data in this manuscript are presented as mean ± SD.

## RESULTS

### MSC EV Characterization

The MSC EVs used in this study have been characterized previously by nanoparticle tracking analysis, spectrophotometry, and flow cytometry.^12^ The EVs had a concentration of 1.16 ×10^9^ particles/ml with a size peak at 123 nm and protein content of 160 μg/ml. A subset of the EVs further concentrated for the EV experiments had a protein content of 330 μg/ml. By flow cytometry the EVs were positive for EV-specific markers CD69, CD81 and CD9, MSC markers CD73 and CD90, and negative for negative control markers CD31, CD45, and HLA-DR. In addition, images of the MSC EVs via scanning electron microscopy were obtained, consistent with the particle size demonstrated via nanoparticle tracking analysis **(Supplemental Figure 1)**.

### MSC Conditioned Media and MSC EVs Protect the Pulmonary Endothelial Barrier in Vitro

**Figure 1:**
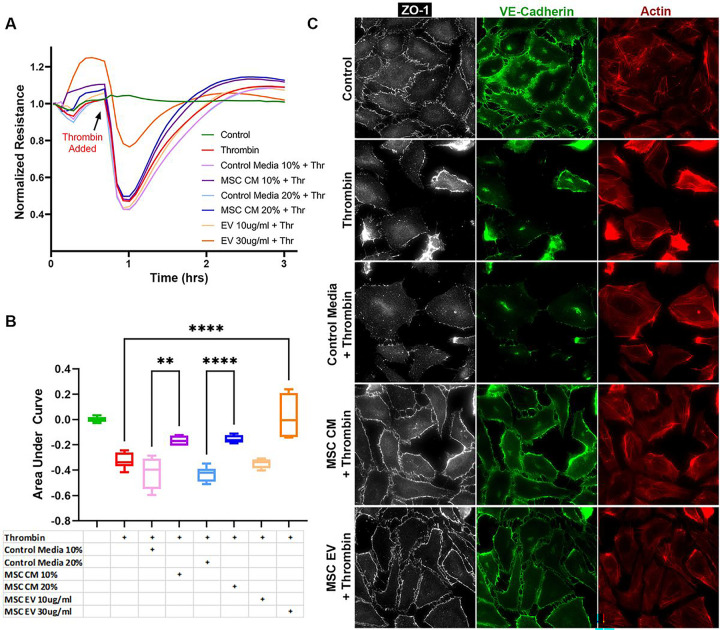
Effects of MSC CM and MSC EVs on a Pulmonary Endothelial Cell Monolayer in Vitro. **(A)**Pulmonary endothelial cell (PEC) monolayer resistance tracings at 4000Hz in response to thrombin challenge. N=4 cell replicates per group. **(B)** Area under the curve (AUC) plot for resistances following the addition of thrombin. **p<.01, ****p<.0001 by one-way ANOVA with post-hoc Tukey’s tests. **(C)**Representative images (40x magnification) of immunofluorescence staining of PEC monolayers for Zonula Occludens-1 (ZO-1, white), VE-cadherin (green), and Actin (red). The addition of thrombin caused decreased resistance across the monolayer as well as loss of ZO-1 and VE-cadherin staining and gap formation with cells. Pre-treatment of the cell monolayers with MSC CM (20%) or MSC EVs (30μg/ml) partially attenuated these effects, whereas MSC control media (20%) was not protective.

Resistance tracings generated by ECIS and AUC calculations demonstrated a drop in resistance across PEC monolayers exposed to thrombin, indicating increased paracellular permeability (Figure 1A, 1B). MSC CM refers to cell culture media containing the MSC secretome, whereas MSC control media has not been exposed to MSCs and thus does not contain MSC-secreted factors or EVs. Cell monolayers that had been pre-treated with MSC CM had significantly higher resistances than those treated with MSC control media at both of the tested doses. The higher dose of MSC EVs at 30 μg/ml was also protective.

Consistent with the ECIS tracings, immunostaining for the tight junction protein ZO-1 and adherens junction protein VE-cadherin demonstrated a loss of ZO-1 and VE-cadherin staining among PEC monolayers exposed to thrombin (Figure 1C). In addition, thrombin exposure resulted in gap formation or separation between cells. Pre-treatment with MSC CM or MSC EVs resulted in relative preservation of PEC barrier integrity as evidenced by attenuation of the loss of ZO-1 and VE-cadherin staining and decreased gap formation. F-actin staining of the PEC monolayer showed reduced stress fiber activation, when compared to the thrombin challenged group.

### MSC Conditioned Media but not MSC EVs Protect the Intestinal Epithelial Barrier in Vitro

**Figure 2:**
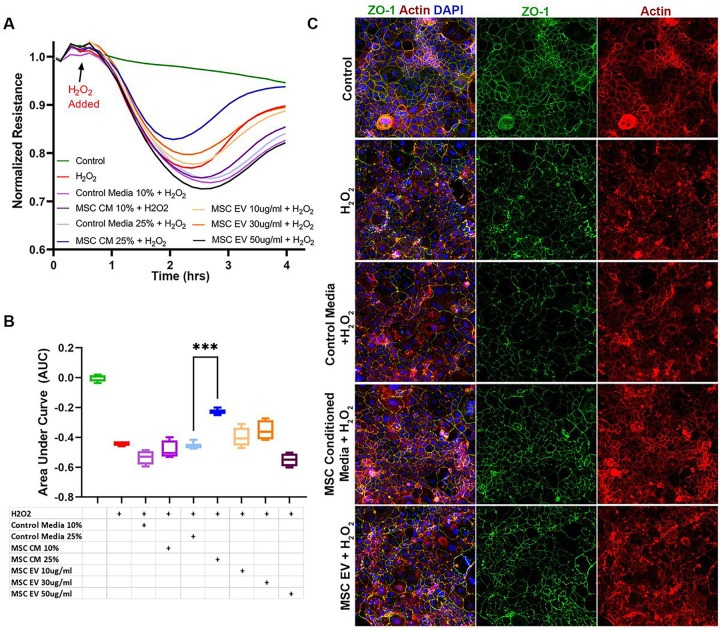
Effects of MSC CM and MSC EVs on the Caco-2 Intestinal Epithelial Cell Monolayer in Vitro. **(A)** Caco-2 intestinal epithelial cell resistance tracings at 1000Hz in response to H2O2 challenge. N=4 cell replicates per group. **(B)** Area under the curve (AUC) plot for resistances following the addition of H_2_O_2_. ***p<.001 by one-way ANOVA with post-hoc Tukey’s tests. **(C)** Representative images (10x magnification) of immunofluorescence staining of Caco-2 monolayers for Zonula Occludens-1 (ZO-1, green), Actin (red), and DAPI (blue). The addition of H_2_O_2_ caused decreased resistance across the monolayer as well as relative loss of ZO-1 staining and actin redistribution. MSC CM (25%) pre-treatment decreased the effects of H2O2, whereas treatment with MSC control media (25%) or MSC EVs (30 μg/ml) were not protective.

Caco-2 intestinal epithelial cell monolayers exhibited decreased resistance or increased permeability in response to H_2_O_2_ exposure (Figure 2A, 2B). MSC CM was significantly protective compared to control media at the higher dose (25%) tested. However, MSC EV pre-treatment was not protective at any of the tested doses. Immunostaining was also performed on the Caco-2 monolayers to evaluate ZO-1 and actin (Figure 2C). H_2_O_2_ exposure resulted in re-distribution of actin filaments and decreased ZO-1 staining compared to control conditions. Pre-treatment with MSC CM preserved actin organization and attenuated the loss of ZO-1. However, similar to the results demonstrated by ECIS, MSC EV pre-treatment did not affect ZO-1 or actin staining.

### MSCs and MSC EVs Preserve Endothelial Adherens Junctions in the Intestine and Lung in Vivo

**Figure 3:**
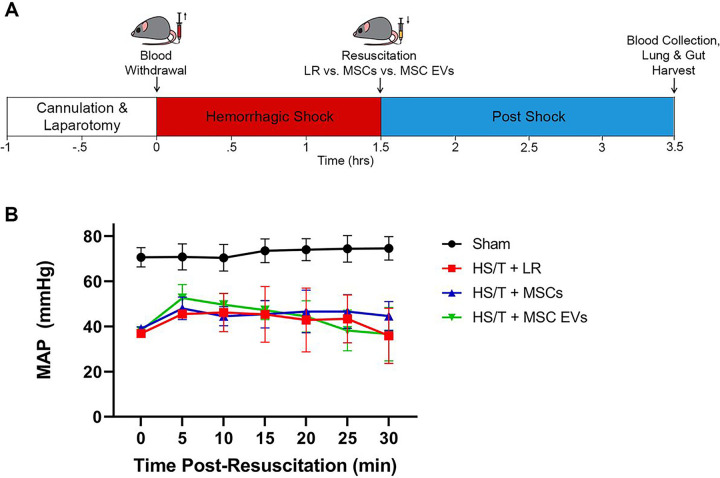
Hemorrhagic Shock and Trauma Mouse Model. (A) Depiction of the 90-minute HS/T model. Mice were resuscitated with LR, MSCs, or MSC EVs (N=5 mice per group). Blood collection and organ harvest were performed 2 hours after the end of shock. (B) Mean arterial pressures (MAPs) during the 30 minutes post-resuscitation. There was no effect of resuscitation group on MAPs (p=0.74) by two-way ANOVA with repeated measures.

**Figure 4:**
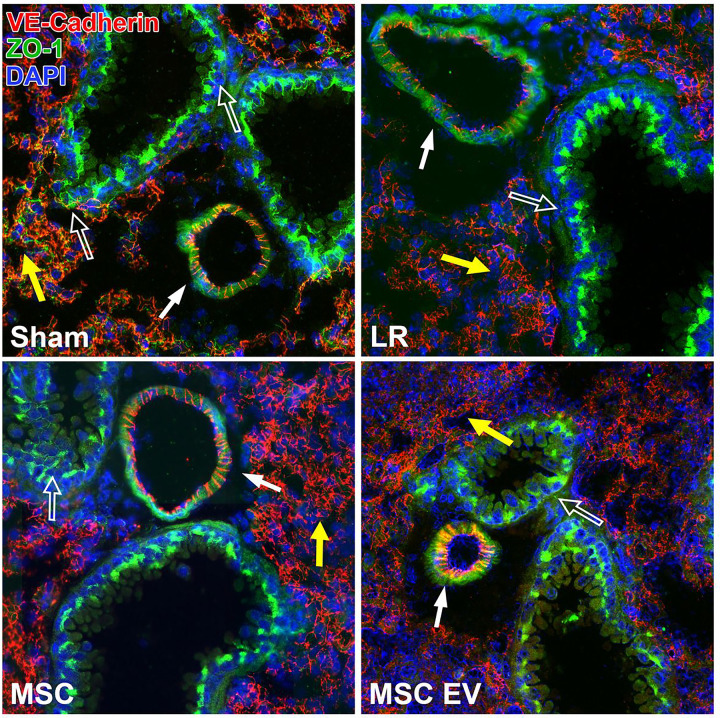
Intercellular Junction Staining in the Lung after Hemorrhagic Shock and Trauma (HS/T). Representative images (20x magnification) of lungs stained for VE-cadherin (endothelial adherens junctions; red), ZO-1 (tight junctions; green), and DAPI (nuclei; blue). Mice subjected to HS/T and resuscitated with LR demonstrate loss of ZO-1 staining between epithelial cells in the airways as well as in the alveolar epithelium. LR-resuscitated mice also demonstrate a loss of staining for VE-cadherin in small blood vessels. Treatment with MSCs or MSC EVs restored VE-cadherin staining suggesting a protective effect on endothelial barrier integrity. MSC treatment also partially protected against loss of ZO-1 in the epithelial-lined airways. *Open arrow* points to airway. *Closed arrow*points to blood vessel. *Yellow arrow* points to alveoli containing capillaries and epithelial cells.

**Figure 5:**
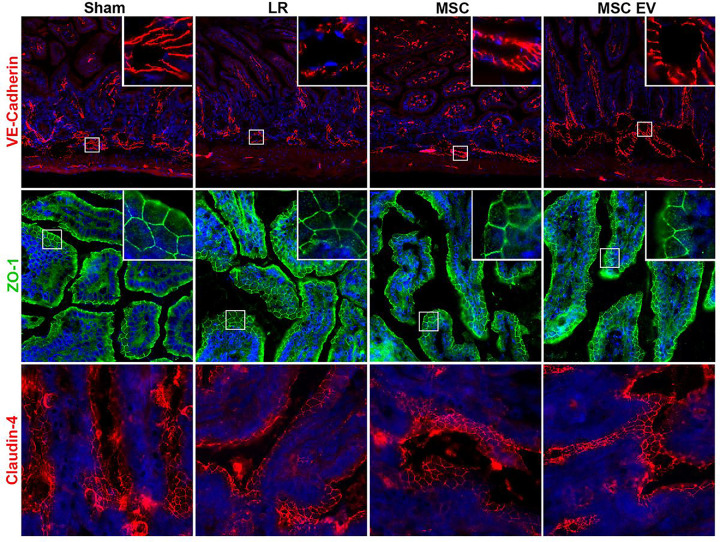
Intercellular Junction Staining in the Small Intestine after Hemorrhagic Shock and Trauma (HS/T). Representative images of small intestine stained for VE-cadherin (adherens junctions; red; 10x magnification), ZO-1 (tight junctions; green; 20x magnification), claudin-4 (tight junctions; red; 20x magnification), and DAPI (nuclei; blue). LR-treated mice demonstrated a loss of VE-cadherin in the submucosal blood vessels (see 40x image inset) of the small intestine, whereas MSC or MSC EV treatment attenuated this finding. Epithelial barrier integrity was evaluated with ZO-1 and claudin-4 staining, however no differences were evident between groups.

The HS/T model schematic is depicted in Figure 3. Mean arterial pressures (MAPs) were similar among mice resuscitated with LR, MSCs, or MSC EVs. Sections of the lungs and small intestine of mice subjected to HS/T were stained to evaluate endothelial and epithelial barrier integrity *in vivo*. In the lungs, HS/T with LR resuscitation induced a qualitative loss of VE-cadherin (adherens junction) staining among endothelial-lined blood vessels and alveolar capillaries compared to sham mice. MSC and MSC EV treatment resulted in relative preservation of VE-cadherin staining. Moreover, there was notable compromise of ZO-1 (tight junction) staining in the epithelial-lined airways that underwent HS/T compared to sham mice, however this finding was partially mitigated in the MSC group. There was also loss of ZO-1 staining in the alveoli regardless of treatment group after HS/T (Figure 4). In the small intestine, sections stained for VE-cadherin demonstrated a loss of VE-cadherin staining in the submucosal vessels after HS/T in LR-treated mice, whereas MSC and MSC EV treatment qualitatively preserved VE-cadherin staining in these vessels. Staining for ZO-1 and claudin-4 was also performed to evaluate epithelial integrity, however, there were no substantial differences across the sham or injured groups (Figure 5).

### Plasma Biomarker Analysis

**Figure 6:**
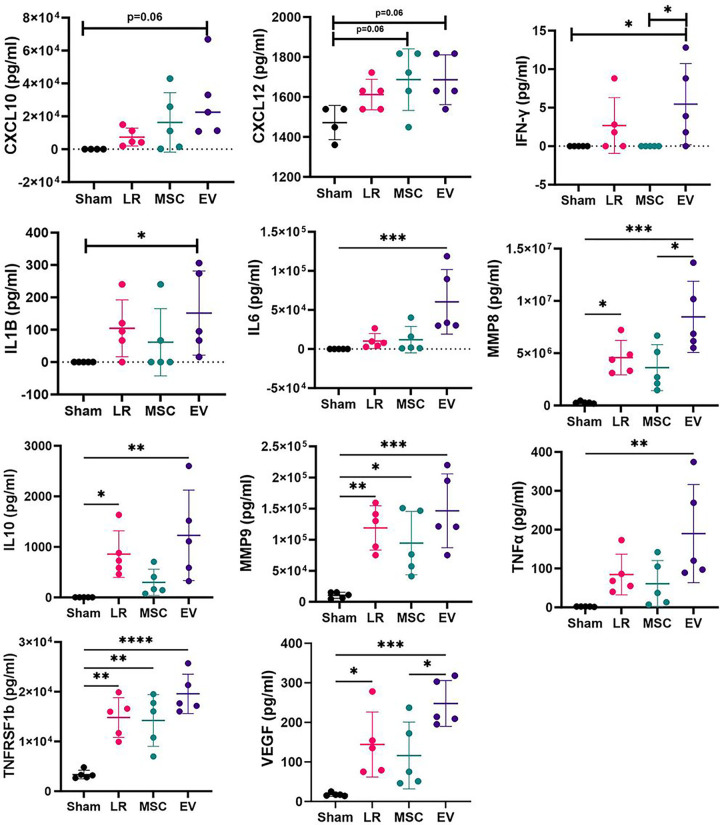
Plasma Biomarker Analysis. Plasma biomarkers of inflammation and endothelial function were evaluated using a custom Luminex^®^ assay (N=5 mice per group). Only biomarkers with significant differences between groups are shown here. Groups were compared using one-way ANOVA for normally distributed data and Kruskal-Wallis testing for data that were not normally distributed. Outliers were defined by the GraphPad program and were removed from analysis. Measurements are expressed in pg/ml.

The levels of each plasma inflammatory biomarker are listed in Table 1. Biomarkers that showed significant differences between any of the groups are depicted in Figure 6. Compared to sham mice, LR-treated mice had significantly higher levels of IL-10, MMP-8, MMP-9, TNFRSF1b, and VEGF, MSC-treated mice had higher levels of TNFRSF1b and showed a trend toward higher levels of CXCL12 (p=0.06), and EV-treated mice had higher levels of IFN-y, IL-1B, IL-6, IL-10, MMP-8, MMP-9, TNFa, TNFRSF1b, and VEGF and showed a trend toward higher levels of CXCL10 (p=0.06) and CXCL12 (p=0.06). Other biomarkers were similar across groups. Notably, both MMP-8 and VEGF were significantly elevated among the LR- and EV-treated groups compared to sham mice, whereas these biomarker levels were not significantly different between MSC-treated mice and sham mice.

## CONCLUSIONS

Our previous work has shown that MSC EVs recapitulate the ability of MSCs to mitigate histologic injury and vascular permeability in the lungs and small intestine in mice after HS/T. This study further demonstrates that MSCs and MSC EVs may target the EOT via preservation of the vascular endothelial barrier early after injury.

In this study, we demonstrated *in vitro* that both MSCs and MSC EVs preserve barrier properties of a pulmonary endothelial monolayer. While MSC CM also helped to maintain barrier integrity of a Caco-2 intestinal epithelial monolayer, MSC EVs were not protective at multiple tested doses. This finding may be due to higher concentrations of protective factors or additional soluble factors in the MSC CM compared to the MSC EVs. *In vivo* in a mouse model of HS/T, both MSCs and MSC EVs attenuated the loss of endothelial adherens junction staining in blood vessels in the lung and small intestine. MSCs but not MSC EVs resulted in a partial protective effect on epithelial tight junctions in the lung. Epithelial tight junctions in the small intestine were not clearly affected in this model. Altogether these results suggest that one potential therapeutic mechanism of MSCs and MSC EVs in HS/T is an early protective effect on vascular barrier integrity.

In addition, at two hours post resuscitation, we did find that there were several differences in the inflammatory biomarker profiles between groups. For example, both MMP8 and VEGF were significantly elevated among LR- and EV-treated (but not MSC-treated) mice compared to sham. MMP8 is known to decreased expression of tight junction proteins in endothelial cells;^22^ and VEGF is a potent vascular permeabilizing agent via multiple mechanisms including phosphorylation and loss of tight and adherens junctions, induction of matrix metalloproteinase expression, and negative regulation of pericyte function.^23^ This suggests that there may be some differences in the immunomodulatory effects of MSCs and MSC EVs after HS/T. There were no significant differences seen across groups, however, for a number of the tested biomarkers. It is possible that other time points than the one used in this study may better capture the immunomodulatory effects of MSCs and MSC EVs in this model, as MSCs and MSC EVs have been shown to have numerous immune-regulating effects in a variety of inflammatory diseases.^24,25^ In addition, others have shown initial systemic immune activation after MSC infusion in mice followed by reduced immune reactivity, further suggesting additional time points would be beneficial to understanding MSC and MSC EV immunomodulation after HS/T.^26^

MSCs and MSC EVs contain numerous bioactive factors, including mRNAs, microRNAs, proteins, and lipids, a combination of which may underly the vasculoprotective properties seen in this study. For example, MSCs and MSC EVs contain the mRNA angiopoietin-1 (Ang-1), a ligand for the Tie2 receptor tyrosine kinase which forms an important endothelial signaling pathway that inhibits vascular permeability and leukocyte-endothelium interactions.^21,27,28^ MSC EVs also contain a number of other factors such as hepatocyte growth factor (HGF), tissue inhibitor of metalloproteinase 3 (TIMP3), and sphingosine 1-phosphate (S1P), which have all been shown to have important roles in maintaining or restoring endothelial barrier function.^28–30^ EVs from MSCs exposed to culture conditions mimicking an ischemic microenvironment have also been found to contain an abundance of proteins involved in pathways including vascular wall cell surface interactions, cadherin signaling, cytoskeletal signaling, and vasculogenesis.^31^

There are several important limitations to this study. First, the Caco-2 cell line used *in vitro* is derived from colon adenocarcinoma, which may not entirely reflect the response of the small intestine to oxidative stress *in vivo*. However, the Caco-2 cell line in culture resembles enterocytes lining the small intestine, is used extensively to study the intestinal epithelial barrier, and is considered the *in vitro* gold standard for the assessment of drug permeability and absorption.^32^ Second, human rather than mouse MSCs and MSC EVs were used in a mouse model, which raises the possibility of an interspecies effect. This was done to best evaluate the product that would be given to humans (i.e. human MSCs and MSC EVs). MSCs are also considered immune evasive and have been used in this manner in other studies.^33^ Third, because there were no changes in tight junction staining in the intestinal epithelial barrier *in vivo*, we were unable to determine the impact of these therapies on the gut barrier. This was an unexpected finding given the clear decrease in pulmonary epithelial tight junction staining after HS/T, and others have demonstrated loss of intercellular junction integrity in the gut in similar models.^34–36^ Finally, a number of the plasma inflammatory biomarkers were unexpectedly not different between sham animal and the shocked groups, though this may not be surprising given that the sham animals in this study do undergo bilateral femoral artery cannulation and ligation, and therefore some degree of hindlimb ischemia, as well as 2 hours of anesthetic time.

In conclusion, this study demonstrates that both MSCs and MSC EVs help to maintain vascular barrier integrity *in vitro* and *in vivo* in a mouse model of HS/T. MSC EVs may therefore be able to recapitulate many of the potential therapeutic benefits of MSCs in HS/T in a cell-free manner, overcoming some of the logistical barriers and disadvantages of live cell administration in patients. MSC-based therapies are currently being evaluated in a variety of clinical settings, with published trials demonstrating positive results and a favorable safety profile.^37^ However, understanding the potential role of these therapies in HS/T is only in its infancy.^9,38^ A number of questions remain to be addressed, including cell source, dosing, timing of delivery, and the duration of their effects. Further studies are also needed to validate MSC-based therapies in critically ill patients with systemic inflammation and coagulopathy and in patients who are receiving simultaneous therapies such as blood products and hemostatic agents.

## Figures and Tables

**Table 1.** Plasma Inflammatory Biomarkers after Hemorrhagic Shock and Trauma.

Analyte	Sham (pg/ml)	LR (pg/ml)	MSC (pg/ml)	EV (pg/ml)
**Ang2**	27324±3424	23371±3809	30293±6566	24191±5852
**CXCL10**	0±0	7379±5459	16318±18089	28938±23111
**CXCL12**	1472±85.73	1612±75.6	1687±154^a^	1687±124.7^a^
**ICAM-1**	15294±4666	12057±3535	12936±2642	14130±2570
**IFN-γ**	0±0	2.68±3.627	0±0	5.46±5.26^a,b^
**IL-1β**	0±0	104.4±88.06	61.4±104	151.6±130^a^
**IL2**	0±0	0.6±1.34	0.72±1.61	2.04±2.82
**IL4**	330±7.97	324.8±27.58	323.8±36.41	331±23.01
**IL6**	53.2±35.32	10198±9560	11992±16983	60396±41279^a^
**IL6Ra**	10349±909.3	10780±639	11144±1209	10399±1529
**IL10**	3.6±0.55	857.4±461.1^a^	299±259.8	1229±895.8^a^
**MMP8**	282150±103143	4577903±1649162^a^	3615807±2199024	8471390±3408584^a,b^
**MMP9**	10488±4969	118897±35588^a^	94408±51062^a^	146319±59425^a^
**Syndecan-1**	8031±3599	9799±1811	12253±3066	10605±2461
**TNF-α**	1.8±0.45	84.4±52.34	60.8±59.78	189.8±126.3^a^
**TNFRSFIa**	1075±272.6	5002±3029	4065±2327	5060±3407
**TNFRSFIb**	3347±854.7	14819±3996^a^	14213±5198^a^	19580±3965^a^
**TNFRSF3b**	5920±978	5506±802	6253±1194	7120±2088
**VEGF**	17.6±4.34	144.2±82.35^a^	116.2±84.53	247.8±57.9^a,b^

Luminex multiplex measurement of plasma from mice at 2 hours post-resuscitation. Groups were compared using one-way ANOVA for normally distributed data and Kruskal-Wallis testing for data that were not normally distributed. Outliers were defined by the GraphPad program and were removed from analysis. Numbers are mean±SD, N=5 mice per group.

a Statistically significant difference compared to sham

b Statistically significant difference compared to MSC. P values as indicated in Figure 6.

## Data Availability

The datasets used and/or analyzed during the current study are available from the corresponding author on reasonable request.
